# The risk of diabetic renal function impairment in the first decade after diagnosed of diabetes mellitus is correlated with high variability of visit-to-visit systolic and diastolic blood pressure: a case control study

**DOI:** 10.1186/s12882-017-0514-9

**Published:** 2017-03-22

**Authors:** Chi-Hsiao Yeh, Hsiu-Chin Yu, Tzu-Yen Huang, Pin-Fu Huang, Yao-Chang Wang, Tzu-Ping Chen, Shun-Ying Yin

**Affiliations:** 1 Department of Thoracic and Cardiovascular Surgery, Chang Gung Memorial Hospital, 222 Mai-Chin Road, Keelung, 204 Taiwan, Republic of China; 2 College of Medicine, Chang Gung University, Tao-Yuan, Taiwan, Republic of China; 3 Department of Nursing, Chang Gung Memorial Hospital, Keelung, Taiwan, Republic of China; 4 Division of Thoracic & Cardiovascular Surgery, Chang Gung Memorial Hospital, 222 Mai-Chin Road, Keelung, 204 Taiwan

**Keywords:** Blood pressure control, Chronic kidney disease, Electronic medical record, Hypertension

## Abstract

**Background:**

The variability of visit-to-visit (VVV) in systolic blood pressure (SBP) and diastolic blood pressure (DBP) is proved as a predictor of renal function deterioration in patients with non-diabetic chronic kidney disease. The purpose of this study was to investigate the relationship of the variability in SBP and the magnitude of renal function impairment for normal renal function patients in the first 10-years diagnosed with type II diabetes mellitus (DM).

**Methods:**

We retrospectively reviewed the electronic medical records of 789 patients who were first diagnosed with diabetes mellitus during 2000–2002 and regularly followed for 10 years with a total of 53,284 clinic visits. The stages of Chronic Kidney Disease (CKD) of every patient were determined using estimated glomerular filtration rate. The occurrence of nephropathy was defined in those patients whose CKD stages elevated equal or larger than three.

**Results:**

Patients were categorized according to the VVV of systolic and diastolic BP into three groups. Patients with high VVV of both SBP and DBP had a 2.44 fold (95% CI: 1.88–3.17, *p* < 0.001) increased risk of renal function impairment compared with patients with low VVV of both SBP and DBP. Risk of renal function impairment for patients with high VVV of either SBP or DBP had a 1.43-fold increase (95% CI: 1.08–1.89, *p* = 0.012) compared with patients with low VVV of both SBP and DBP. Cox regression analysis also demonstrated that every 1-year increase of DM diagnosed age significantly raised the risk of renal function impairment with a hazard ration of 1.05 (95% CI: 1.04–1.06, *p* < 0.001).

**Conclusions:**

Not only VVV of SBP but also VVV in DBP is correlated with diabetic nephropathy in the first decade for patients diagnosed with type 2 DM.

## Background

Diabetes mellitus (DM) is the primary cause of end stage renal disease [1]. In adults aged 18 years or older with DM, 71% are reported to have hypertension [1], a major risk of microvascular complications and cardiovascular mortality [2]. In patients with DM, several risk factors including mean blood pressure (BP), albuminuria, high hemoglobin A1c and serum cholesterol have been shown to accelerate the progression of chronic kidney disease (CKD) [3, 4].

In diabetic patients with CKD, the decline of the glomerular filtration rate (GFR) is highly variable, ranging from 2 to 20 mL/min/year [3]. The risk factors for losing filtration power, such as hypertension, proteinuria, glycemic control and lipids, have not been studied extensively. Controversy existed as some of these factors contributed to renal function impairment in diabetic patients [5]. To identify the risk factors of renal function deterioration is important for development of prevention modalities in diabetic patients’ treatment. In clinical diabetic treatment guideline [6], absolute BP is used as a therapeutic target to prevent clinical stroke and heart disease, as well as CKD with paucity of evidence [7].

Recently, the visit-to-visit variability (VVV) of systolic BP (SBP) has been shown to be a novel risk factor for development of renal function decline in non-diabetic CKD [8], progression of albuminuria and nephropathy in patients with type II DM [9, 10], and deterioration of renal function for stage 3–4 diabetic CKD patients [4]. Although it is widely known that average blood pressure is related to renal function deterioration. However, little is known about the long-term association of VVV of SBP and diastolic BP (DBP) with renal function impairment in patients with normal renal function at the diagnosis of DM. The association between VVV of BP and CKD generally consider SBP measurements at a few time points and in a short to medium follow-up period, limiting the appreciation of the full impact of SBP and DBP on CKD. BP fluctuation across long periods and its effect on renal function impairment in diabetic patients with normal renal function are typically not considered. Therefore, we evaluated the long-term relationship between the VVV of SBP and DBP and the change of the CKD stage in patients from the beginning of diagnosed with type 2 DM.

## Methods

### Patients and study design

We retrospectively collect the 10-year measurements of blood pressure, body weight, body height, and laboratory datas at every outpatient clinic visit of 789 patients who were first diagnosed with type 2 DM during 2000–2002 at Chang Gung Memorial Hospital, Keelung. Type 2 DM was diagnosed in accordance with the criteria of American Diabetes Association [11]. Body mass index (BMI) was defined as weight (kilograms) divided by height (meters) squared. Patients were classified as nonsmokers, former smokers, or current smokers according to the electronic medical record. Patients with advanced renal dysfunction (serum Cr more than 2.0 mg/dL) before diagnosed with DM were excluded from this study. Cardiovascular disease (CVD) included coronary artery disease or myocardial infarction, and ischemic stroke or transient ischemic attack [12] that resulted from atherosclerosis after type II diabetes was diagnosed. The coronary artery disease was confirmed by coronary angiography and the ischemic stroke or transient ischemic attack was confirmed by computed tomography or clinical symptoms. The definition of dyslipidemia was either total cholesterol >200 mg/dL, low density lipoprotein cholesterol >100 mg/dL, low density lipoprotein cholesterol <50 mg/dL in female and <40 mg/dL in male, or triglyceride >150 mg/dL which were based on the standards of the laboratory in our hospital. Hypertension was defined as systolic pressure ≥130 mmHg or diastolic pressure ≥ 80 mmHg in diabetic patients [13].

We then evaluated relationships of variability in blood pressure to change of CKD stage during the 10-year follow-up period. The study was conducted in accordance with the Declaration of Helsinki and was approved by the Institutional Review Board of Chang Gung Memorial Hospital; informed consent was waived. Blood pressure measurements at every outpatient clinic visit throughout the follow-up period were recorded. Fasting serum total cholesterol, low-density lipoprotein, high-density lipoprotein, and triglyceride concentrations were assessed using standard enzymatic methods. Hemoglobin A_1c_ was assayed using high-performance liquid chromatography and expressed with the unit defined by the National Glycohemoglobin Standardization Program.

### Definition of BP variability

Throughout the 10-year consecutive visits from the beginning of the observation period, the mean office BP and the VVV of SBP and DBP (expressed as within-individual standard deviation (SD) were calculated. The BP instability indice was expressed as the delta BP, which was defined as a difference between the maximum and the minimum BP, through all 10-year visits [14].

### Definition of CKD and renal function impairment

Serial serum creatinine data were collected and eGFR was determined by the abbreviated CKD Epidemiology Collaboration equation [15]. CKD was defined as a decreased estimated glomerular filtration rate (eGFR) (<60 ml/min per 1.73 m2) for 3 months. CKD stage was defined in accordance with the guideline of National Kidney Foundation [16], which stage 1, 2, 3, 4, and 5 had a eGFR of ≥ 90, 60 to 90, 30 to 59, 15–29, and < 15 ml/min per 1.73 m^2^ or commencement of dialysis therapy, respectively. Renal function impairment was defined as two or more CKD stages (from stage 1 or 2 to stage 3–5 or 4–5, respectively) deterioration without recovery.

### Statistical analysis

Means and frequencies of potential confounding variables were calculated. The relationships between variability in SBP and DBP, as well as other variables, and renal function impairment were examined by Pearson’s correlation analyses. To examine the effects of various factors on the deterioration of renal function, the following factors were considered simultaneously as independent variables for Cox multiple regression analysis: age of DM diagnosed, sex, BMI, average SBP and DBP, SD of SBP and DBP, hemoglobin A1c, total cholesterol, triglyceride, smoking status, presence of CVD, hypertension and dyslipidemia. All continuous variables are presented as the mean ± SD or absolute number. A *P* value <0.05 was considered statistically significant. The area under each receiver operating curve (ROC) and 95% confidence intervals (CI) were estimated to compare the relative ability of SD of SBP and DBP to identify risk of renal function impairment in diabetic patients. Optimal cut-off points for SD of SBP and DBP indicator were determined [17]. The collinearity among average SBP and DBP, SD of SBP and DBP, and delta SBP and DBP was estimated using variance inflation factor [18].

## Results

Eight hundred and twenty-five patients were first diagnosed with DM from 2000 to 2002. Thirty-six patients who were died or loss of follow-up were excluded. None of these patients died from renal failure. The characteristics of the 789 patients, who were first diagnosed with DM from 2000 to 2002 and followed for 10 years, enrolled in this study are shown in Table 1. The total number of measurements of BP, BMI, HbA_1c_, lipid profile, and serum creatinine throughout 10- year of data collection was 49739, 35432, 27424, 9327, and 14123, respectively. The characteristics of the study patients were shown in Table 1. The overall mean age of the patients diagnosed with DM was 53.3 ± 10.5 years. At baseline, the mean initial serum creatinine was 0.93 ± 0.45 mg/dL, the mean initial eGFR was 88.6 ± 22.7 mL/min per 1.73 m2, and the mean office SBP and DBP was 136.6 ± 10.1 and 73.5 ± 6.3 mm Hg, respectively. The median observation period was 4451 ± 453 days. At the end of the observation period, the mean serum creatinine level was 1.10 ± 0.81 mg/dL and the mean eGFR was 75.1 ± 27.6 mL/min per 1.73 m2. The 10 year mean change of CKD stage was 1.2 ± 0.8.

**Table 1:** Patients’ demographic and clinical characteristics

Patients (n)	789
Age at diabetes diagnosis (years)	53.3 ± 10.5
Sex (male/female)	373/416
Smoking (none/former/current)	598/45/146
Hypertension (%)	597 (75.7)
Hyperlipidemia (%)	758 (96.1)
Body mass index (kg/m^2^)	26.8 ± 3.9
Mean number of measurements	45.0 ± 24.5
Mean SBP (mmHg)	137 ± 10
Mean number of measurement	63.0 ± 28.9
SD of SBP (mmHg)	14.7 ± 3.6
Delta SBP (mmHg)	71.7 ± 25.0
Mean DBP (mmHg)	73.5 ± 6.3
SD of DBP (mmHg)	7.4 ± 2.0
Delta DBP (mmHg)	37.7 ± 14.3
Hemoglobin A1c (%) (mmol/mol)	7.6 ± 1.0 (60.0 ± 10.9)
Mean number of measurements	34.8 ± 10.9
Total cholesterol (mg/dL)	193.4 ± 28.6
Mean number of measurements	11.9 ± 5.8
High-density lipoprotein (mg/dL)	38.5 ± 10.7
Low-density lipoprotein (mg/dL)	118.7 ± 20.3
Triglyceride (mg/dL)	149.8 ± 113.8
Initial eGFR (mL/min/1.73 m2)	88.6 ± 22.7
Mean number of measurements	17.9 ± 6.7
Final eGFR (mL/min/1.73 m2)	75.1 ± 27.6
Clinical Events during10-year follow-up	
CVD ^a^ (%)	115 (14.6)
Interval from diabetes diagnosis (years)	5.2 ± 3.1
Change in CKD stage	1.2 ± 0.7
Renal function impairment (%)	309 (39.2)
CKD stage 4 or 5 (%)	83 (10.5)
Total follow-up period (days)	4451 ± 453

*Abbreviations*: *SBP* systolic blood pressure, *DBP* diastolic blood pressure, *SD* standard deviation, *eGFR* estimated *glomerular filtration rate*, *CVD* cardiovascular disease, *CKD* chronic kidney disease

^a^Defined as coronary artery disease or myocardial infarction, and ischemic stroke or transient ischemic attack

Cox regression analyses revealed that the SD of SBP was positively correlated with the occurrence of renal function impairment (*P* < 0.001, Hazard ratio (HR) = 1.063, 95% CI = 1.028–1.100), as well as the SD of DBP (*P* < 0.024, HR = 1.081, 95% CI = 1.010–1.156). The age of DM first diagnosed had also positively correlated with the occurrence of renal function impairment after 10-year follow-up (*P* < 0.001, HR = 1.048, 95% CI = 1.036–1.060). Our results found that maximum, minimum or delta of SBP and DBP had no significant independent correlations between renal function impairment after 10-year of DM diagnosed. And multiple regression analysis demonstrated that other factors, such as mean or SD of hemoglobin A_1c_, BMI, total cholesterol, low-density lipoprotein, high-density lipoprotein, triglyceride, were not independently correlated with the occurrence of renal function impairment, as shown in Table 2.

**Table 2:** Multivariate Cox regression analyses of renal function impairment in 789 patients after 10-year diabetes diagnosis

Independent variable	β	*P* Value
Sex (female = 0)	0.250	0.081
Age of DM diagnosed	0.047	<0.001
Non-smoking		
Former smoker	0.004	0.990
Current smoker	0.098	0.560
Hypertension	0.687	0.001
Dyslipidemia	0.119	0.772
Mean SBP	0.023	0.435
SD of SBP	0.062	<0.001
CV of SBP	2.414	0.944
Delta of SBP	−0.005	0.227
Mean DBP	−0.048	0.269
SD of DBP	0.077	0.024
CV of DBP	−0.047	0.856
Delta of DBP	0.002	0.787
Mean BMI	0.84	0.047
SD of BMI	−0.293	0.119
Mean HbA1c	−0.167	0.251
SD of HbA1c	0.214	0.485
Mean serum cholesterol	0.004	0.482
SD of serum cholesterol	−0.008	0.111
Mean serum LDL	−0.013	0.053
SD of serum LDL	−0.001	0.860
Mean serum HDL	0.024	0.357
SD of serum HDL	0.006	0.726
Mean serum triglyceride	−0.004	0.047
SD of serum triglyceride	0.002	0.509

*Abbreviations*: *HbA1c* hemoglobin A1c, *SBP* systolic blood pressure, *DBP* diastolic blood pressure, *SD* standard deviation, *CV* coefficient of variation, *BMI* body mass index, *LDL* low density lipoprotein, *HDL* high density lipoprotein

For clinical application, we calculated the area under the ROC curves for the SD of SBP (0.87 ± 0.02) and DBP (0.85 ± 0.03) and categorized patients into high or low SD of SBP or DBP. The best cut-point BP was calculated based on the Youden Index [19], which was calculated as sensitivity + specificity − 1. Cut-off points of SD of SBP and DBP, where sensitivity approximates specificity for renal function impairment, are 16.3 and 7.6 mmHg, respectively. Patients with SD of SBP and DBP higher than the cut-off values were defined as high VVV of SBP and DBP, respectively. Patients were grouped as low VVV of SBP and DBP, high VVV of SBP or DBP, and high VVV of SBP and DBP. The characteristics of patients in these three groups were shown in Table 3. Using univariate analysis, the age of DM diagnosed, hypertension history, BMI, mean SBP and DBP, SD of SBP and DBP, delta SBP and DBP, and mean hemoglobin A1c, and initial eGFR were significantly different between these three groups of patients.

**Table 3:** Demographic and clinical characteristics compared between patients with low VVV of SBP *and* DBP, high VVV of SBP *or* DBP, and high VVV of SBP *and* DBP

	Low VVV of SBP *and* DBP group (*n* = 370)	High VVV of SBP *or* DBP group (*n* = 241)	High VVV of SBP *and* DBP group (*n* = 178)	*P*
Age at diabetes diagnosis (years)	52.8 ± 10.1	51.5 ± 10.4	56.6 ± 10.6	*<0.001*
Sex (male/female)	172 (46.5)	120 (49.8)	81 (47.3)	0.629
Smoking (none/former/current)	287/17/66	179/18/44	132/10/36	0.594
Hypertension (%)	237 (64.1)	199 (82.6)	161 (90.4)	*<0.001*
Hyperlipidemia (%)	358 (96.8)	227 (94.2)	173 (97.2)	0.191
Body mass index (kg/m^2^)	26.4 ± 3.6	27.3 ± 4.2	27.0 ± 3.9	*0.007*
Mean SBP (mmHg)	133.9 ± 9.6	137.3 ± 9.2	141.2 ± 10.6	*<0.001*
Mean number of measurements	60.4 ± 26.0	63.8 ± 29.9	67.8 ± 32.6	*0.019*
SD of SBP (mmHg)	12.3 ± 2.0	14.9 ± 2.6	19.3 ± 2.8	*<0.001*
Delta SBP (mmHg)	59.3 ± 15.1	73.3 ± 23.0	95.2 ± 26.6	*<0.001*
Mean DBP (mmHg)	71.6 ± 5.5	75.3 ± 5.9	74.8 ± 7.2	*<0.001*
SD of DBP (mmHg)	6.0 ± 0.9	8.1 ± 1.4	9.6 ± 1.7	*<0.001*
Delta DBP (mmHg)	29.4 ± 6.7	41.7 ± 13.4	49.5 ± 16.6	*<0.001*
HbA1c (%) (mmol/mol)	7.4 ± 0.9 (57.0 ± 9.8)	7.8 ± 1.0 (62.0 ± 10.9)	7.7 ± 1.1 (61.0 ± 12.0)	*<0.001*
Mean number of measurements	36.1 ± 10.8	34.5 ± 10.5	32.3 ± 11.2	*0.001*
Total cholesterol (mg/dL)	191.8 ± 26.7	193.0 ± 27.0	197.1 ± 33.9	0.132
Mean number of measurements	12.1 ± 5.9	11.9 ± 5.7	11.2 ± 5.8	0.220
High-density lipoprotein (mg/dL)	38.9 ± 10.6	38.1 ± 10.8	38.4 ± 10.7	0.678
Low-density lipoprotein (mg/dL)	118.3 ± 19.2	119.3 ± 20.9	118.8 ± 21.6	0.832
Triglyceride (mg/dL)	141.0 ± 117.4	155.6 ± 90.3	160.1 ± 132.4	0.119
Initial eGFR (mL/min/1.73 m2)	81.4 ± 26.0	77.5 ± 25.9	59.0 ± 27.0	*0.003*
Mean number of measurements	17.4 ± 6.4	17.6 ± 6.4	19.3 ± 7.5	*0.005*
Final eGFR (mL/min/1.73 m2)	88.6 ± 26.6	76.0 ± 26.6	55.9 ± 27.6	*<0.001*
Clinical Events during10-year follow-up				
CVD ^a^ (%)	28 (7.6)	43 (17.8)	44 (24.7)	*<0.001*
Interval from diabetes diagnosis (years)	5.8 ± 2.9	5.1 ± 2.7	4.8 ± 3.5	0.425
Change in CKD stage	1.0 ± 0.6	1.1 ± 0.6	1.5 ± 0.8	*<0.001*
Renal function impairment (%)	110 (29.3)	92 (36.8)	125 (62.8)	*<0.001*
Interval from diabetes diagnosis (days)	3899 ± 1214	3710 ± 1285	3079 ± 1468	*<0.001*
CKD stage 4 or 5 (%)	35 (9.3)	29 (11.6)	22 (11.1)	0.620
Interval from diabetes diagnosis (days)	4366 ± 620	4311 ± 676	4272 ± 730	0.267
Total follow-up (days)	4488 ± 400	4424 ± 474	4413 ± 521	0.102

*Abbreviations*: *HbA1c* hemoglobin A1c, *SBP* systolic blood pressure, *DBP* diatolic blood pressure, *SD* standard deviation, *eGFR* estimated *glomerular filtration rate*, *CVD* cardiovascular disease, *CKD* chronic kidney disease

^a^Defined as coronary artery disease or myocardial infarction, and ischemic stroke or transient ischemic attack

All significant change with *p*<0.05 had been italicized

**Fig. 1:**
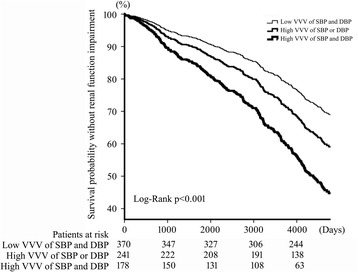
Kaplan-Meier plot of renal function impairment in newly diagnosed type II diabetic patients for 10-year follow-up. Patients was grouped into low VVV of SBP and DBP, high VVV of SBP or DBP, and high VVV of SBP and DBP

After 10 years of DM diagnosis, the patients with high VVV of both SBP and DBP had the highest percentage of peripheral artery disease, cerebrovascular disease, coronary artery disease or myocardial infarction, transient ischemic attack or stroke, and the highest percentage of patients with renal function impairment, which all were significantly different among these three groups. Cox multivariate regression revealed that only the age of DM diagnosed and the group of VVV of SBP and DBP were significant risk factors for development of renal function impairment after 10-year follow-up, as shown in Table 4. All the variance inflation factors among mean, SD and delta of SBP and DBP were less than three, which excluded the collinearity between these factors. The risk of renal function impairment in patients with high VVV of both SBP and DBP significantly increased 2.773 fold (*p* < 0.001, 95% CI = 2.128–3.612) compared that of patients with low VVV of both SBP and DBP. Whereas the risk of renal function impairment in patients with wither high VVV of SBP or DBP increased 1.587 fold (*p* = 0.001, 95% CI = 1.195–2.107) compared that of patients with low VVV of both SBP and DBP. The renal function intact survival curve for these three groups of patients was shown in Fig. 1.

**Table 4:** Multivariable Cox regression analysis of renal function impairment

	Hazard Ratio	95% CI	*P*
Age at diabetes diagnosis (+1 year)	1.046	1.034–1.058	<0.001
Low VVV of SBP and DBP	1		
High VVV of SBP or DBP	1.587	1.195–2.107	0.001
High VVV of SBP and DBP	2.773	2.128–3.612	<0.001

*Abbreviations*: *SBP* systolic blood pressure, *DBP* diastolic blood pressure, *SD* standard deviation, *CI confidence interval*

## Discussion

This study showed that VVV of both SBP and DBP were significantly associated with the change to CKD stage in the first decade of patients diagnosed with DM, whereas mean office SBP/DBP, delta SBP/DBP, mean serum lipid profile, mean hemoglobin A_1c_ concentration, and SD of hemoglobin A_1c_ concentration were not correlated with the occurrence of renal function impairment. To the best of our knowledge, this is the first study to explore the association of the VVV of SBP and DBP and renal function decline in the first decade of patients with DM.

In the present study, not only the VVV of SBP was significantly associated with the change of CKD stage, but the VVV of DBP was also significantly associated with the occurrence of renal function impairment in our study patients group. For the first time, our result demonstrated that a VVV of SBP higher than 16.3 mmHg or a VVV of DBP higher than 7.6 mmHg would significantly increase the risk of decline of renal function in the first decade of patients diagnosed with DM. Furthermore, with both high VVV of SBP and DBP would even increase the risk of renal function impairment to 2.773 fold compared with those patients with both low VVV of SBP and DBP.

Our results are in accordance with other study findings, which showed a significant association between VVV of BP and progression of nondiabetic CKD [8, 9]. However, in contrast with our findings, the other study with a small sample size (69 patients) and short study period (32 months) from Yokota et al. reported negative association between VVV of BP and renal function deterioration in CKD patients [4]. The other large cohort study included 114,900 adults with CKD stage 3–4 followed for 180 days revealed that the highest quintile of the SD would increase the risk of end-stage renal disease by 1.45-fold (CI 1.02–2.05) [20].

Many studies proved that an increase in VVV of SBP was one of the factors which may contribute to the progression of renal function deterioration [9, 10]. Mancia et al. suggested that a steeper rate of blood pressure oscillations, in addition to blood pressure levels, was related to end-organ damage in hypertensive patients [21]. In hypertensive individuals, large arteries lose their compliance and became stiff in hypertensive patients which resulted in less buffering of blood pressure changes [22] and wider blood pressure oscillation for any given change in the stroke volume, which would be detected with the fluctuations of blood pressure [23]. The initiation and progression of atherosclerosis of the renal vasculature, resulted from the oscillatory shear stress, could contribute to the impairment of renal function [24]. However, these studies did not prove that high VVV of DBP might be another factor result in the renal function impairment in DM patients. Our 10-year long-term follow-up results revealed that high VVV of DBP had detrimental effects on the renal function of diabetic patients. The impact of DBP in renal function ad been proved by many studies. Brazy et al. stressed that reduced DBP, instead of SBP or mean BP, to less than 90 mmHg could slow down the rate of renal function deterioration [25]. Wight et al. reported a positive correlation was observed between proteinuria and DBP [26]. The discrepancy of our results from the other VVV-related studies might come from the duration of the study design. Most related reports included diabetic patients in CKD stage 3 or higher and followed less than 5 years. Our study included intact renal function patients who were in the first decade of DM diagnosed. The long-term period of our study might be the most important factor that made high VVV of DBP stand out as a risk factor of renal function impairment.

The retrospective nature of the present study and the sample size are two of the limitations of the present study. The possibility of type 2 error existed. Other limitations are the standardized procedure of blood pressure measurement and the medication prescription record through 10-year follow-up period. Certain classes of antihypertensive regimens, such as non-β-blocker-based and non-rennin-angiotensin system-based [27] or calcium channel blockers [28], had been prove in reduction of the VVV of blood pressure with preservation of end-organ damage in hypertensive patients. The anti-hypertensive drugs in each patient were not consistent throughout 10 years. It is very difficult to clarify the effect of VVV amelioration of each category of anti-hypertensive drugs, such as calcium channel blockers, which had been proved to blunt the association between the VVV of BP and renal function decline [4, 28]. Another limitation of the present study is the absence of data regarding antihypertensive prescription fill data and patients’ adherence to medication regimens. However, low antihypertensive medication adherence explained only a small proportion of VVV of BP [29], which implied that the absence of medication adherence data does not have a major impact on the result of the present study.

Nevertheless, this study has several strengths including the one-decade follow-up of patients initially diagnosed with DM with CKD and available information on demographic, clinical, and long-term BP data. In addition, the use of the electronic medical record database provided real-world evidence on the status of hypertension control in the first decade renal function prognosis of diabetic patients and minimizes selection bias related to self selection into the study. Our results prove that VVV of SBP and DBP has significant prognostic value. However, prospective investigations in stabilization of VVV of SBP and DBP are in need to identify the optimal therapeutic strategies and potentially modify the clinical practice in hypertensive patients with type II DM.

## Conclusions

In conclusion, the present study showed, in patients with intact renal function, significant association between high VVV of SBP and DBP with renal function decline in the first decade of DM diagnosed.

## Abbreviations

BMI: Body mass index
BP: Blood pressure
CKD: Chronic kidney disease
CVD: Cardiovascular disease
DBP: Diastolic blood pressure
eGFR: estimated Glomerular filtration rate
GFR: Glomerular filtration rate
HbA1c: Hemoglobin A1c
HR: Hazard ratio
SBP: Systolic blood pressure
SD: Standard deviation
VVV: Visit-to-visit variability
